# New Secodaphnane-Type Alkaloids with Cytotoxic Activities from *Daphniphyllum angustifolium* Hutch

**DOI:** 10.1007/s13659-021-00309-w

**Published:** 2021-05-11

**Authors:** Qing-Yun Lu, Jia-Hui Zhang, Ying-Yao Li, Xue-Xue Pu, Cui-Shan Zhang, Shuai Liu, Jia-Jia Wan, Ying-Tong Di, Xiao-Jiang Hao

**Affiliations:** 1 State Key Laboratory of Phytochemistry and Plant Resources in West China, Kunming Institute of Botany, Chinese Academy of Sciences, Kunming, 650201 People’s Republic of China; 2 School of Life Sciences, Southwest University, Chongqing, 400715 China; 3 Yunnan University, Kunming, People’s Republic of China; 4 Yunnan University of Traditional Chinese Medicine, Kunming, People’s Republic of China; 5 University of Chinese Academy of Sciences, Beijing, 100049 People’s Republic of China

**Keywords:** *Daphniphyllum angustifolium* hutch, Secodaphnane-type, Daphnioldhanol A, Cytotoxic activity

## Abstract

One new *Daphniphyllum* alkaloid, daphnioldhanol A (**1**), together with three known ones, were isolated from the stem part of *Daphniphyllum angustifolium* Hutch. Their structures were elucidated by spectroscopic methods and comparing with the literature data. Compound **2** is a new natural product, but known by synthesis as a racemate. Compound **1** exhibited week cytotoxic activity against Hela cell line with IC_50_ of 31.9 μM.

**Supplementary Information:**

The online version contains supplementary material available at 10.1007/s13659-021-00309-w.

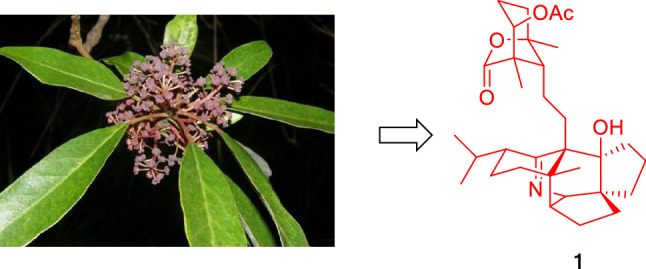

## Introduction

**Fig. 1:**
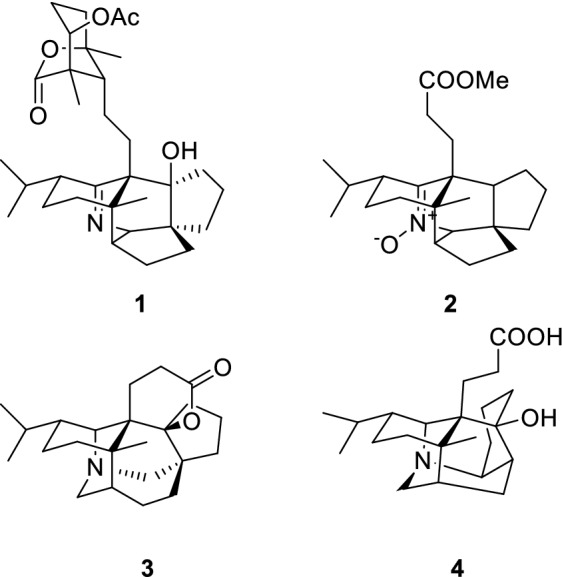
The structures of compounds **1**–**4**

Alkaloids are a class of compounds with significant activities (with a variety of novel skeletons) that are widely found in nature [1–7]. *Daphniphyllum* alkaloids are a structurally diversified group of complex polycyclic natural products isolated from the *Daphniphyllum* genus [8]. Since these unique, versatile and complex nitrogen heterocyclic compounds exhibit a wide range of biological activities and are extremely challenging, they have aroused great interest in total synthesis and biosynthetic studies [9–16]. In recent years, quite a number of new *Daphniphyllum* alkaloids have been isolated and identified, and some of them possessed novel skeletons [17–20]. In our continued search for *Daphniphyllum* alkaloids with interesting skeletons [21–24], one new *Daphniphyllum* alkaloid, daphnioldhanol A (**1**), together with three known ones, were isolated from the stems of *Daphniphyllum angustifolium* Hutch (Fig. 1). Herein, the isolation, structural elucidation, and bioactivities of these compounds are reported.

## Results and Discussion

### Structure Elucidation of the Compounds

Daphnioldhanol A (**1**) was obtained as white amorphous powder. Its molecular formula, C_32_H_48_NO_5_, was established by positive HRESIMS at *m/z* 526.3534 [M+ H]^+^ (calcd 526.3534), with 10 degrees of unsaturation. The IR absorptions implied the presence of hydroxyl (3441 cm^−1^), and an imine moiety (1631 cm^−1^). The ^13^C NMR and DEPT data of **1** displayed 32 carbon signals (Table 1), due to three tetrasubstituted *sp*^2^ carbon atoms at lower field and 29 *sp*^3^ carbon atoms (5 × C, 6 × CH, 11 × CH_2_, 6 × CH_3_) at higher field. According to the molecular formula and relative NMR data, one CH group (*δ*_C_ 65.4, *δ*_H_ 2.95) was ascribed to those bearing an N-atom, while one quaternary C-atom (*δ*_C_ 84.4) were attributed to those bearing an O-atom. Additionally, three *sp*^2^ quaternary carbons were attributable to one lactone carbonyl (*δ*_C_ 180.2), one ester carbonyl (*δ*_C_ 172.2), and one iminium group (*δ*_C_ 168.4), while taking into account the three degrees of unsaturation. The remaining seven degrees of unsaturation were accounted for the presence of the heptacyclic system of **1**.

**Table 1:** ^1^H and ^13^C NMR spectroscopic data for compound **1**^a^ (*δ* in ppm and *J* in Hz)

No.	*δ*_C_	*δ*_H_ (Mult. *J*)	No.	*δ*_C_	*δ*_H_ (Mult. *J*)
1	168.4	–	16a	54.1	3.67 (dt, 14.4, 3.6)
2	40.5	1.47 (o)	16b		2.50 (d, 15)
3a	33.7	2.09 (m)	17a	43	2.89 (m)
3b		1.08 (m)	17b		2.23 (d, 4.2)
4a	38.4	2.06 (m)	18	32.5	1.68 (m)
4b		1.61 (m)	19	21.9	0.96 (d, 6.6)
5	38.7	–	20	22.4	1.02 (d, 6.6)
6	55.2	2.73 (t, 9.0)	21	27.3	1.13 (s)
7	65.4	2.95 (s)	22	58.1	1.86 (m)
8	54.3	–	23	51.6	–
9	84.4	–	24	18.8	1.20 (s)
10	54.2	–	25	180.2	–
11a	35	2.64 (dd 12, 4.8)	26	72.1	4.77 (d, 4.8)
11b		1.10 (m)	27a	26.9	1.85 (o)
12a	30.3	1.85 (o)	27b		1.61 (o)
12b		1.52 (dd, 11.4, 5.4)	28a	27	1.85 (o)
13a	28.2	1.95 (m)	28b		1.61 (o)
13b		1.69 (m)	29	87.7	–
14a	27.9	1.77 (o)	30	24.8	1.47 (o)
14b		1.17 (m)	31	171.2	–
15	44.4	1.77 (o)	32	22	2.13 (s)

^a^Recorded in Methanol-*d*_4_ at 800 MHz (^1^H) and 200 MHz (^13^C)

The ^1^H and ^13^C NMR spectra of **1** were closely related to those of the known compound Daphnioldhanine I [25], with the exception of the loss of signal for a CH in the latter and the addition of signal for quaternary carbon with a hydroxyl (*δ*_C_ 84.4), which were supported by the HMBC correlations of H-11/C-9, H-13/C-9, H-15/C-9, H-16a/C-9, H-17/C-9.

**Fig. 2:**
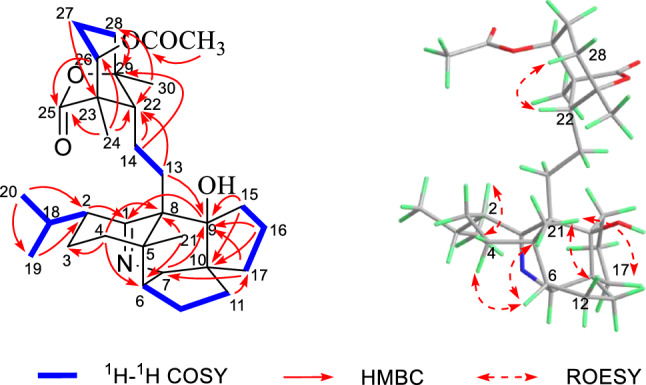
^1^H-^1^H COSY, key HMBC and ROESY correlations of **1**

To determine the orientation of the hydroxyl at C-9, we compared the ^13^C NMR data of both **1** and daphnioldhanine I, which revealed that 9-OH substituent significantly shields the C-21 (5 ppm decrease) in the former. This indicated that the 9-OH in **1** should take a *β*-orientation. Moreover, the remaining relative configuration of **1** was elucidated from ROESY correlations as shown in computer-generated 3D drawing, which was the same as that of the daphnioldhanine I (Fig. 2).

The known compounds were identified as (−)-nitrone 17 (**2**) [26], daphnilactone A (**3**) [27], dapholdhamine B (**4**) [28], respectively, by comparison of their spectroscopic data with those reported in the literature (Fig. 1). Compound **2** was obtained as white amorphous powder. MS analysis of **2** revealed a [2 M + H]^+^ peak at *m/z* 747. By comparison of its ^1^H and ^13^C NMR data with those of (±)-nitrone 17 in the literature, high similarity between them indicated that **2** shared the same structure as the latter. However, the compound **2** is a new chiral natural product with OR at − 31.75°, but known by synthesis is racemate.

**Scheme 1:**
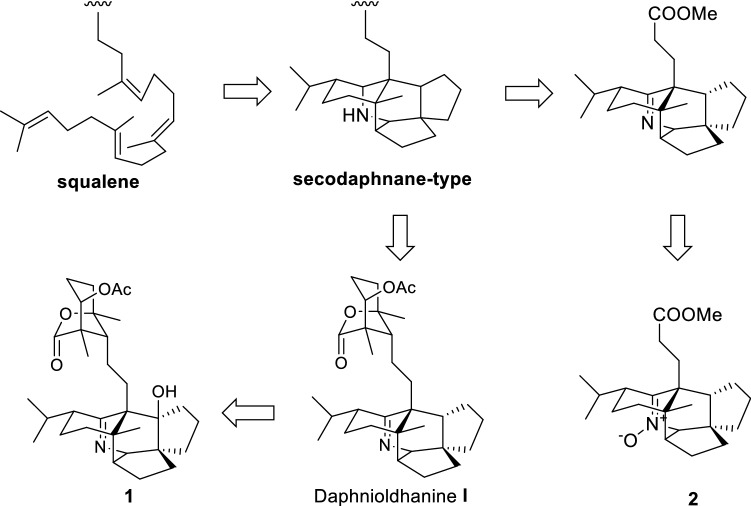
Plausible Biosynthetic Pathway of **1**

A plausible biogenetic pathway for **1** and **2** was proposed as shown in Scheme 1. Biogenetically, both **1** and **2** should be the derivatives of secodaphnane-type alkaloid [13, 29], which might be originated from sequalene, as proposed from Heathcock [26]. Then, **1** and **2** might be formed via different pathway.

### Cytotoxic Activity

Both compounds **1** and **2** have been tested for their cytotoxicity against Hela, MCF-7, A549, MGC-803 and COLO-205 human cancer cell lines in vitro. The results indicated that **1** exhibited weak cytotoxic activity against Hela cell line with IC_50_ of 31.9 μM (Table 2).

**Table 2:** Cytotoxic activity of Compound **1** against Hela, MCF-7, A549, MGC-803 and COLO-205 human cancer cell lines *in vitro*

Human cancer cell lines	IC_50_ (μM)	
	Compound **1**	Doxorubicin
Hela	31.9	0.77
MCF-7	> 76	1.57
A549	52.2	1.92
MGC-803	69.7	1.05
COLO-205	71.8	2.23

## Experimental

### General Experimental Procedures

Optical rotations were measured with a Jasco P-1020 polarimeter. UV spectra were obtained using a Shimadzu UV-2401A spectrophotometer. A Tenor 27 spectrophotometer was used for IR spectra as KBr pellets. 1D and 2D NMR spectra were recorded on Bruker spectrometer with TMS as internal standard. HRESIMS was performed on a triple quadrupole mass spectrometer. Semi-preparative HPLC was performed on an Agilent 1100 liquid chromatograph with a Waters X-Bridge Prep Shield RP18 (10 × 150 mm) column. Column chromatography (CC) was performed using silica gel (100–200 mesh and 300–400 mesh, Qingdao Marine Chemical, Inc., Qingdao, P. R. China) and Sephadex LH-20 (40–70 μm, Amersham Pharmacia Biotech AB, Uppsala, Sweden).

### Plant Material

The stems of *Daphniphyllum Angustifolium* used in this study was collected from Jinfo mountain, Chongqing, P. R. China, in October 2013, and botanically authenticated by professor Deng Hong-ping. A voucher specimen (KIBHAO2014012) was deposited in State Key Laboratory of Phytochemistry and Plant Resources in West China, Kunming Institute of Botany, Chinese Academy of Sciences.

### Extraction and Isolation

Air-dried stems of *Daphniphyllum Angustifolium* (20 kg) were powdered and extracted with MeOH (24 h × 3) at room temperature, and the solvent was evaporated in vacuo. The MeOH extract was then partitioned between EtOAc and TFA/H_2_O at pH 3.0. Water-soluble materials, after being adjusted at pH 10.0 with saturated Na_2_CO_3_, were partitioned with CHCl_3_. CHCl_3_-soluble materials (100.6 g) was subjected to silica gel column chromatography (CC) and eluted with gradient CHCl_3_/MeOH to yield five fractions F-1–F-5. F-4 was repeatedly submitted to silica gel CC and Sephadex LH-20, then purified by HPLC to afford compounds **1** (1.2 mg) and **2** (2.0 mg). Accordingly, **3** (18.0 mg) was obtained from F-1; **4** (2.5 mg) was obtained from F-5.

### Daphnioldhanol A (**1**)

Daphnioldhanol A (**1**): White amorphous powder; C_32_H_47_NO_5_; Positive HR-EI-MS at *m*/*z* 526.3534 [M + H] ^+^ (calcd. for C_32_H_48_NO_5_, 526.3527); [*α*]_D_^20^ =  + 9.88° (*c* = 0.54, MeOH); UV (MeOH) *λ*_max_ (log *ε*) 265 (3.43) nm, 242 (3.56) nm, 215 (3.93) nm; IR: *ν*_max_ (KBr) cm^–1^: 3440, 2928, 2869, 1772, 1743, 1713, 1631, 1383, 1226, 1057, 1028 cm^–1^.

### Nitrone 17 (**2**)

(−)-Nitrone 17 (**2**): Colorless oil; C_23_H_35_NO_3_; ESI-MS (positive): *m/z* 747 [2 M + H] ^+^; ^1^H NMR (CDCl_3_, 400 MHz) *δ*_H_: 3.72 (3H, s), 1.58 (3H, s), 1.02 (1H, d, 6.16), 0.94 (3H, s), 0.85 (3H, d, 6.48); ^13^C NMR (CDCl_3_, 100 MHz) *δ*_C_: 157.2 (C-1), 48.8 (C-2), 27.0 (C-3), 39.0 (C-4), 51.7 (C-5), 52.5 (C-6), 84.1 (C-7), 50.9 (C-8), 52.5 (C-9), 52.9 (C-10), 33.4 (C-11), 22.7 (C-12), 26.2 (C-13), 31.6 (C-14), 25.7 (C-15), 37.0 (C-16), 38.9 (C-17), 31.5 (C-18), 21.0 (C-19), 20.6 (C-20), 23.3 (C-21), 174.1 (C-22), 51.9 (C-23).

### Cytotoxicity Assays

Cytotoxic activity of compound **1** against Hela, MCF-7, A549, MGC-803, and COLO-205 human cancer cell lines in vitro were measured using methylthiazoletetrazolium (MTT) assay [30]. Doxorubicin was used as a positive control.

## Concluding Remarks

In conclusion, one new *Daphniphyllum* alkaloid, daphnioldhanol A (**1**), together with three known ones, were isolated from the stem part of *D. angustifolium* Hutch. Compound **1** exhibited week cytotoxic activity against Hela cell line.

## Supplementary Information

Below is the link to the electronic supplementary material.

Supplementary file1 (DOCX 2262 kb)
