# Supplementary material for: New Secodaphnane-Type Alkaloids with Cytotoxic Activities from Daphniphyllum angustifolium Hutch
Source: Nat Prod Bioprospect. 2021 May 11;11(4):453–7. doi: 10.1007/s13659-021-00309-w (PMC8275811; doi:10.1007/s13659-021-00309-w)

**New** **secodaphnane-type alkaloids with cytotoxic activity from *Daphniphyllum angustifolium* Hutch*.***

Qing-Yun Lu^a, e^, Jia-Hui Zhang^a, b^, Ying-Yao Li^a, c^, Xue-Xue Pu^a, d^, Cui-Shan Zhang^a, e^, Shuai Liu^a, e^, Jia-Jia Wan^a, e^, Ying-Tong Di^a, d^*and Xiao-Jiang Hao^a,^ *

*^a^ State Key Laboratory of Phytochemistry and Plant Resources in West China,*

*Kunming Institute of Botany, Chinese Academy of Sciences, Kunming 650201,*

*PR China*

*^b^ School of Life Sciences, Southwest University, Chongqing, 400715, China*

*^c^* *Yunnan University, Kunming, P.R. China*

*^d^ Yunnan University of Traditional Chinese Medicine, Kunming, P.R. China*

*^e^ University of Chinese Academy of Sciences, Beijing 100049, PR China*

*Corresponding authors.

Tel./fax: +86-0871-65223070. *E-mail addresses*: diyt@mail.kib.ac.cn (Y. T. Di).

Tel./fax: +86-0871-65223070. *E-mail addresses*: haoxj@mail.kib.ac.cn (X. J. Hao).

**Supporting Information**

**Contents:**

S1.1 ^1^H NMR spectrum of Daphnioldhanol A (**1**) in Methanol-*d*_4_

S1.2 ^13^C NMR spectrum of Daphnioldhanol A (**1**) in Methanol-*d*_4_

S1.3 HSQC spectrum of Daphnioldhanol A (**1**) in Methanol-*d*_4_

S1.4 ^1^H-^1^H COSY spectrum of Daphnioldhanol A (**1**) in Methanol-*d*_4_

S1.5 HMBC spectrum of Daphnioldhanol A (**1**) in Methanol-*d*_4_

S1.6 ROESY spectrum of Daphnioldhanol A (**1**) in Methanol-*d*_4_

S1.7 HRESIMS spectrum of Daphnioldhanol A (**1**)

S1.8 UV spectrum of Daphnioldhanol A (**1**) in Methanol

S1.9 IR spectrum of Daphnioldhanol A (**1**)

S1.10 Optical rotation spectrum of Daphnioldhanol A (**1**)

S1.11 ^1^H NMR spectrum of (-)-Nitrone 17 (**2**) in CDCl_3_

S1.12 ^13^C NMR spectrum of (-)-Nitrone 17 (**2**) in CDCl_3_

S1.13 ESIMS spectrum of (-)-Nitrone 17 (**2**)

S1.14 Optical rotation spectrum of (-)-Nitrone 17 (**2**)

S1.1 ^1^H NMR spectrum of Daphnioldhanol A (**1**) in Methanol-*d*_4_


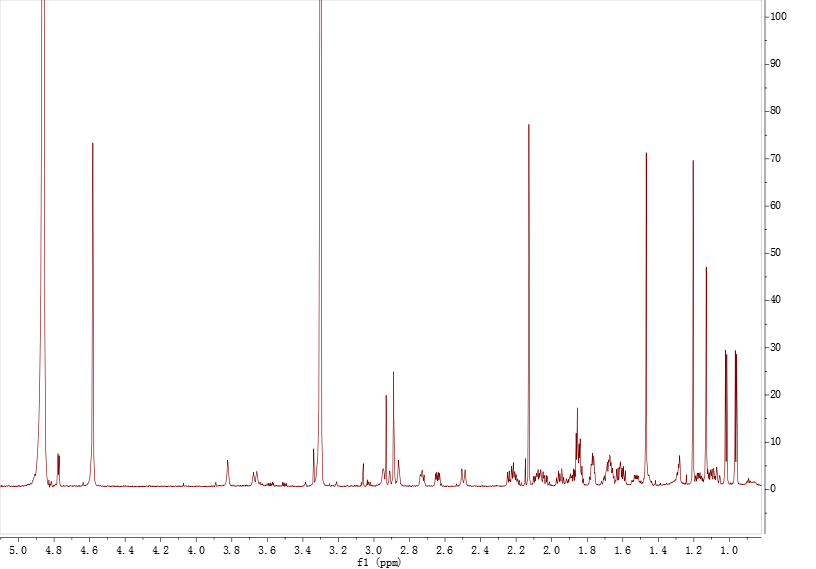


S1.2 ^13^C NMR spectrum of Daphnioldhanol A (**1**) in Methanol-*d*_4_


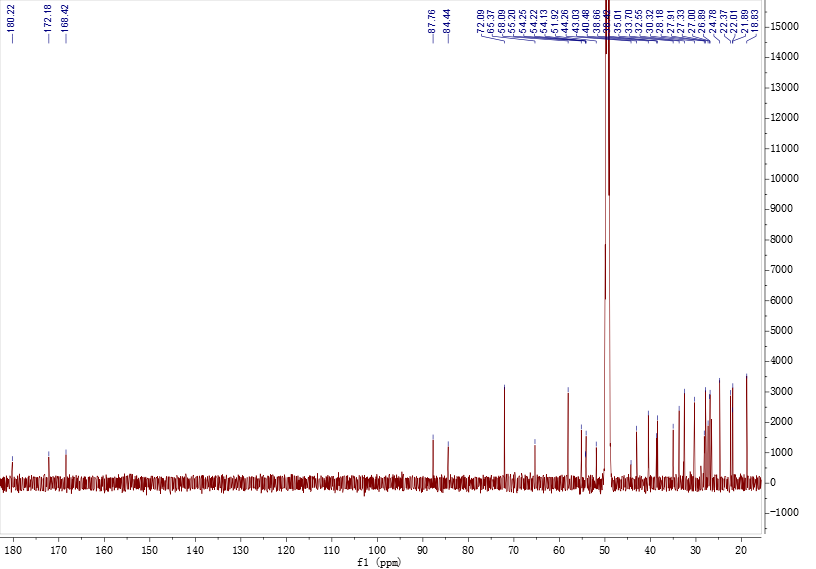


S1.3 HSQC spectrum of Daphnioldhanol A (**1**) in Methanol-*d*_4_


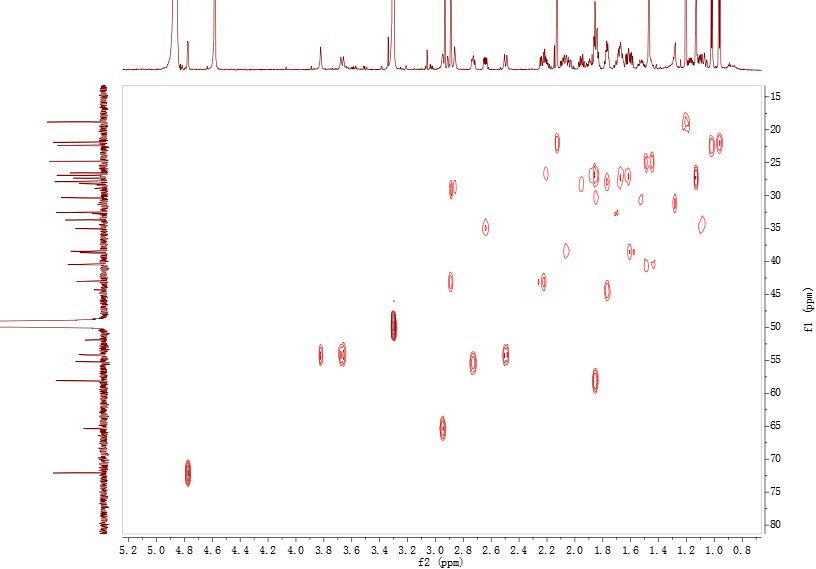


S1.4 ^1^H-^1^H COSY spectrum of Daphnioldhanol A (**1**) in Methanol-*d*_4_


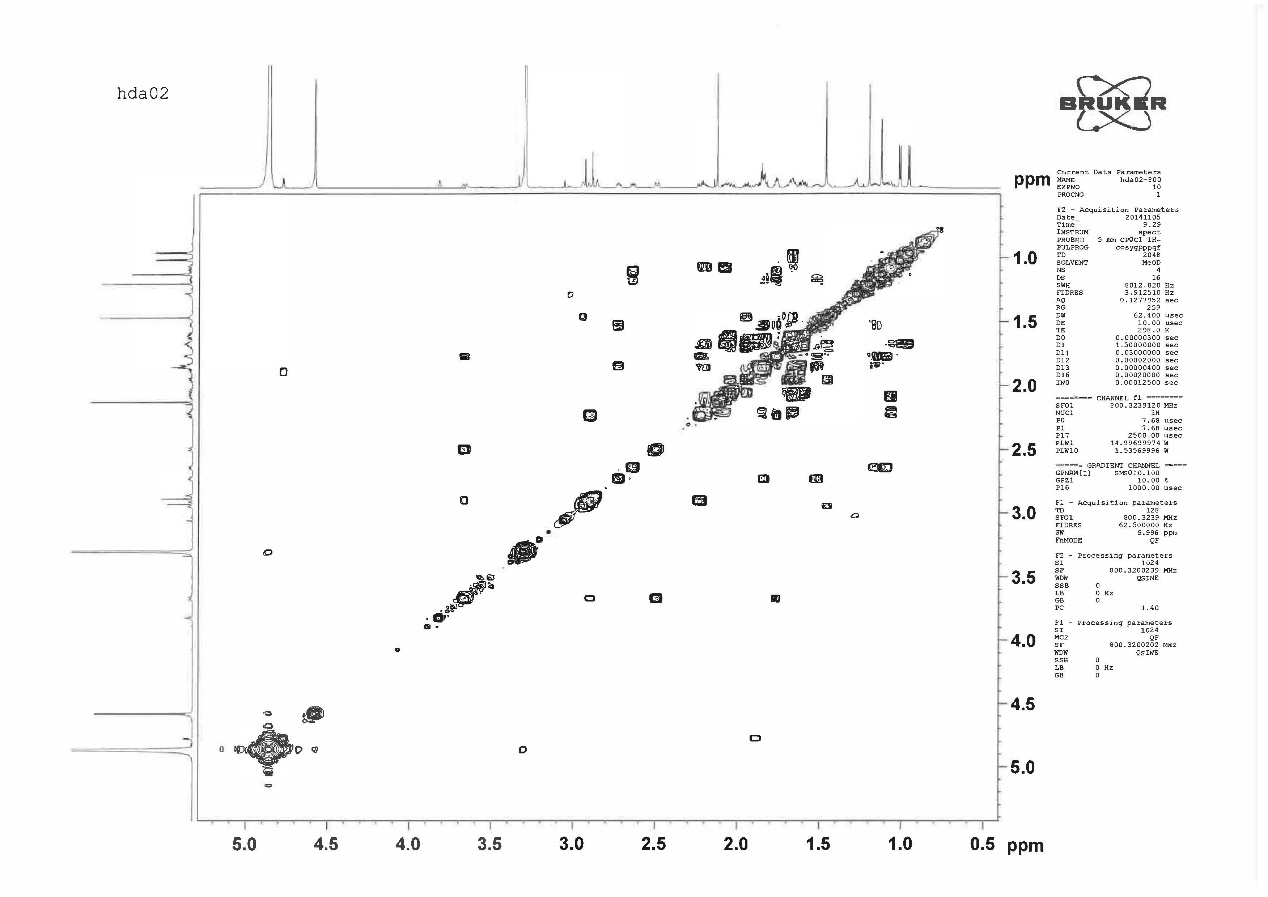


S1.5 HMBC spectrum of Daphnioldhanol A (**1**) in Methanol-*d*_4_


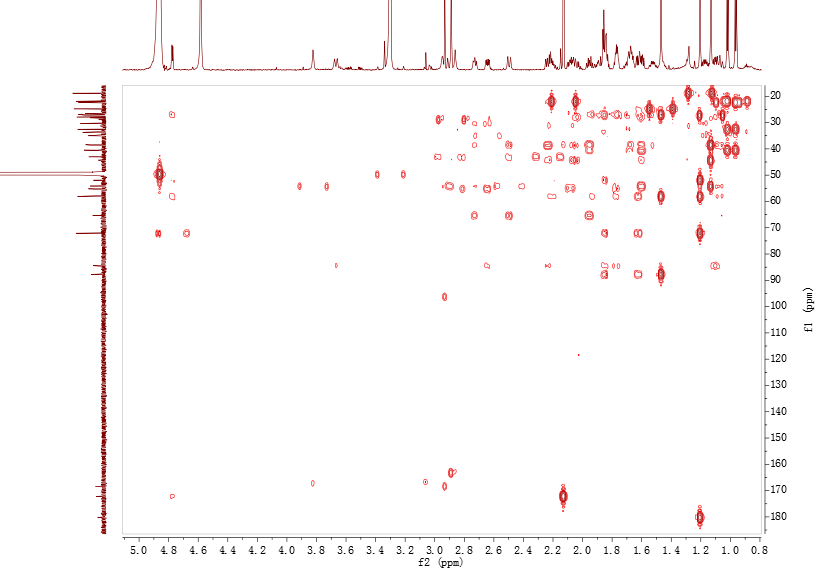


S1.6 ROESY spectrum of Daphnioldhanol A (**1**) in Methanol-*d*_4_


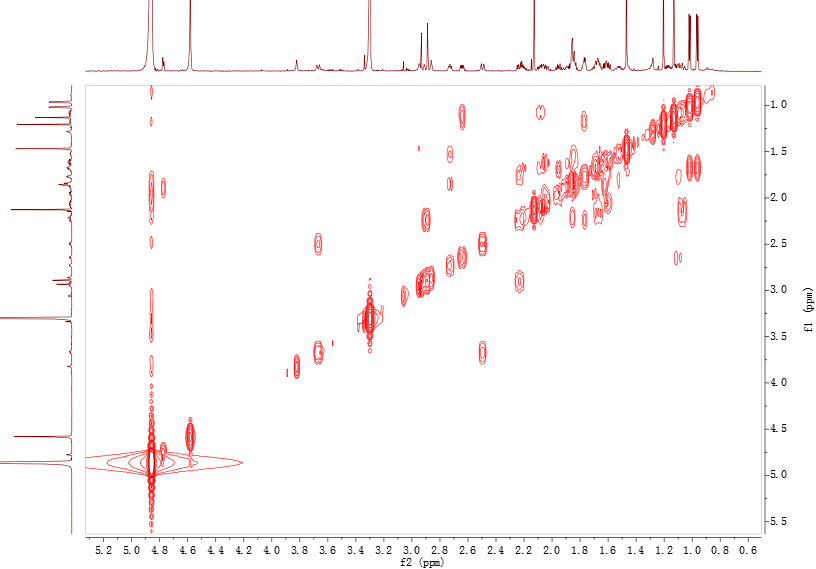


S1.7 HRESIMS spectrums of Daphnioldhanol A (**1**)


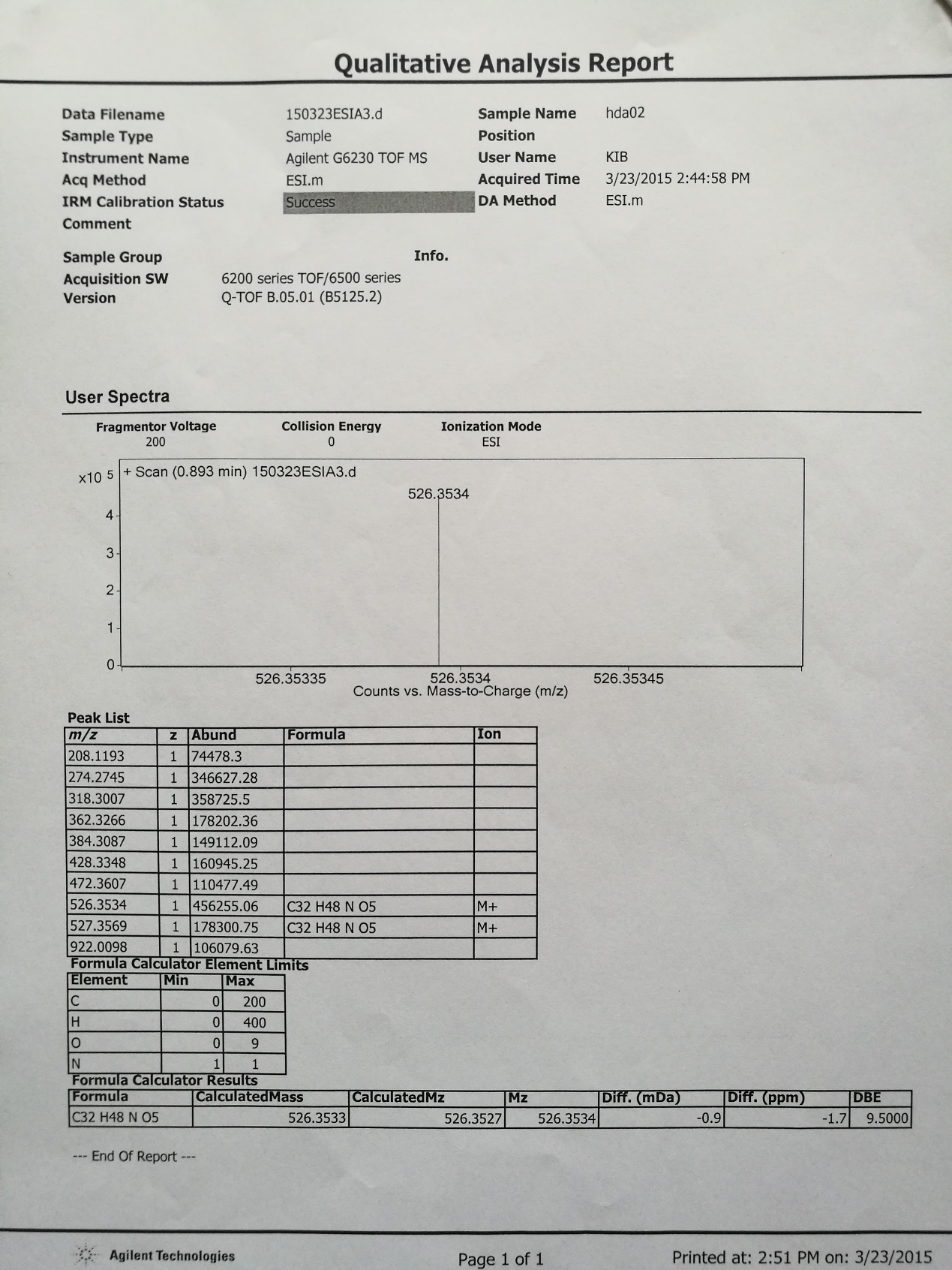


S1.8 UV spectrum of Daphnioldhanol A (**1**) in Methanol


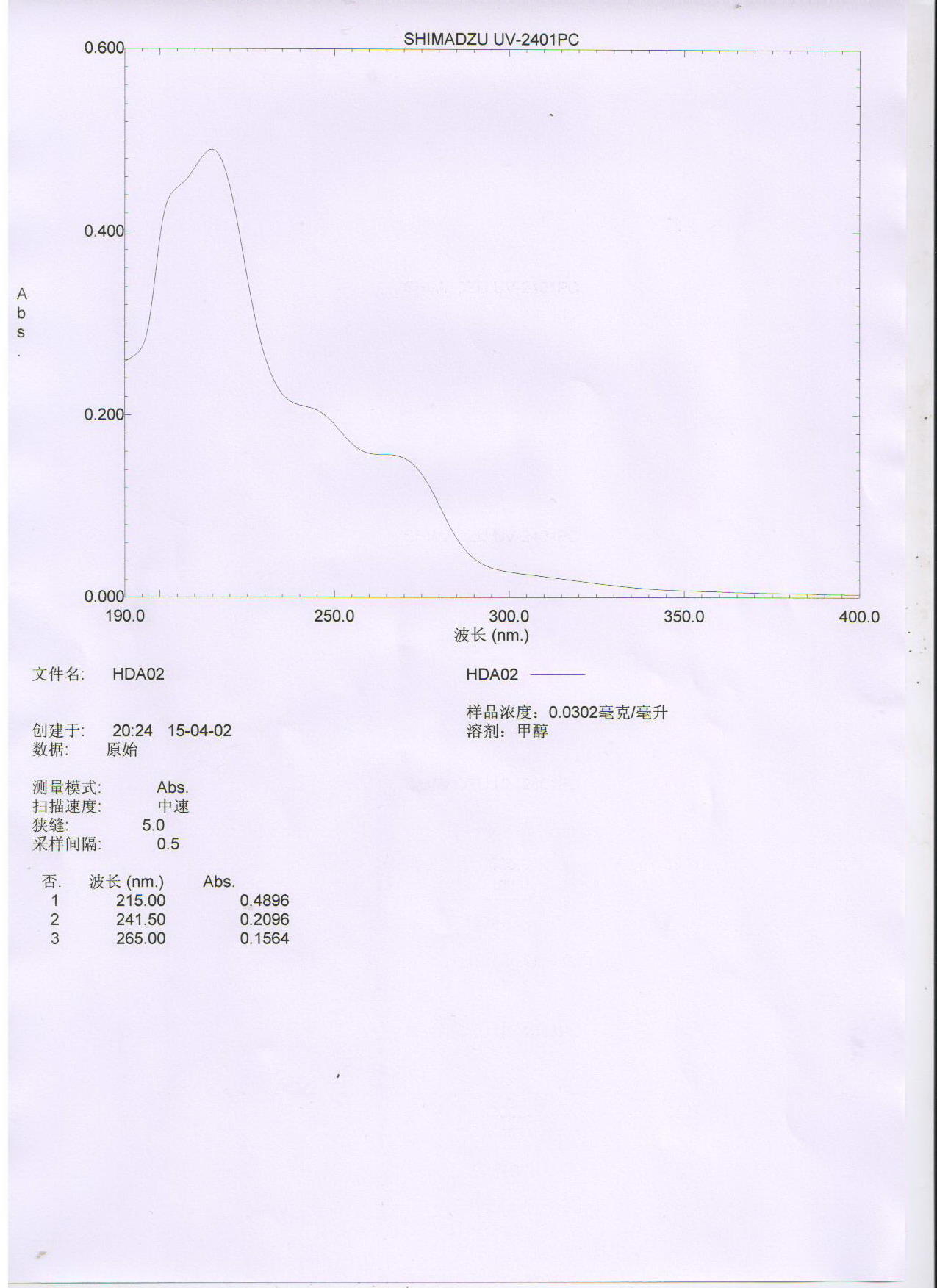


S1.9 IR spectrum of Daphnioldhanol A (**1**)


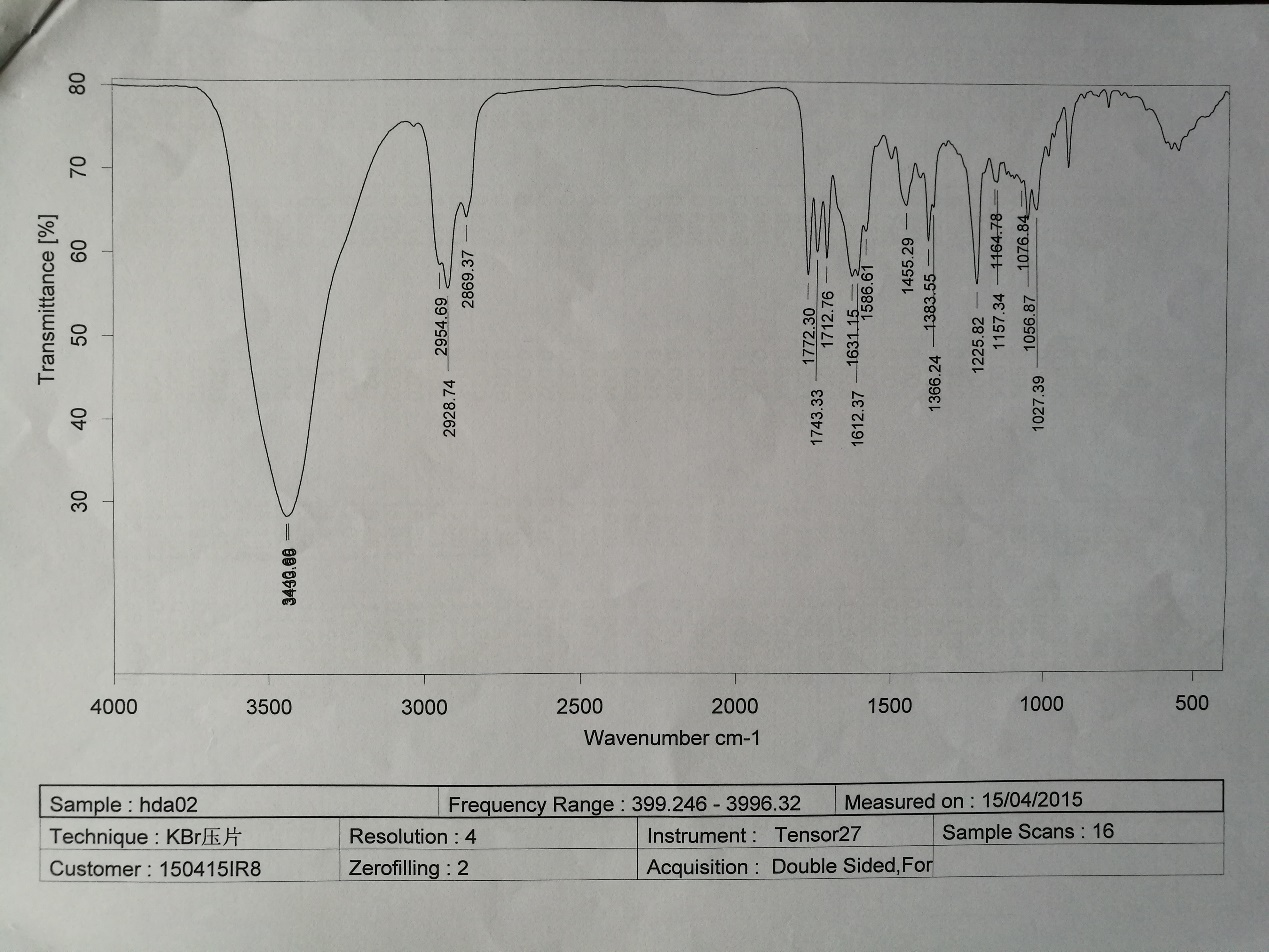


S1.10 Optical rotation spectrum of Daphnioldhanol A (**1**)


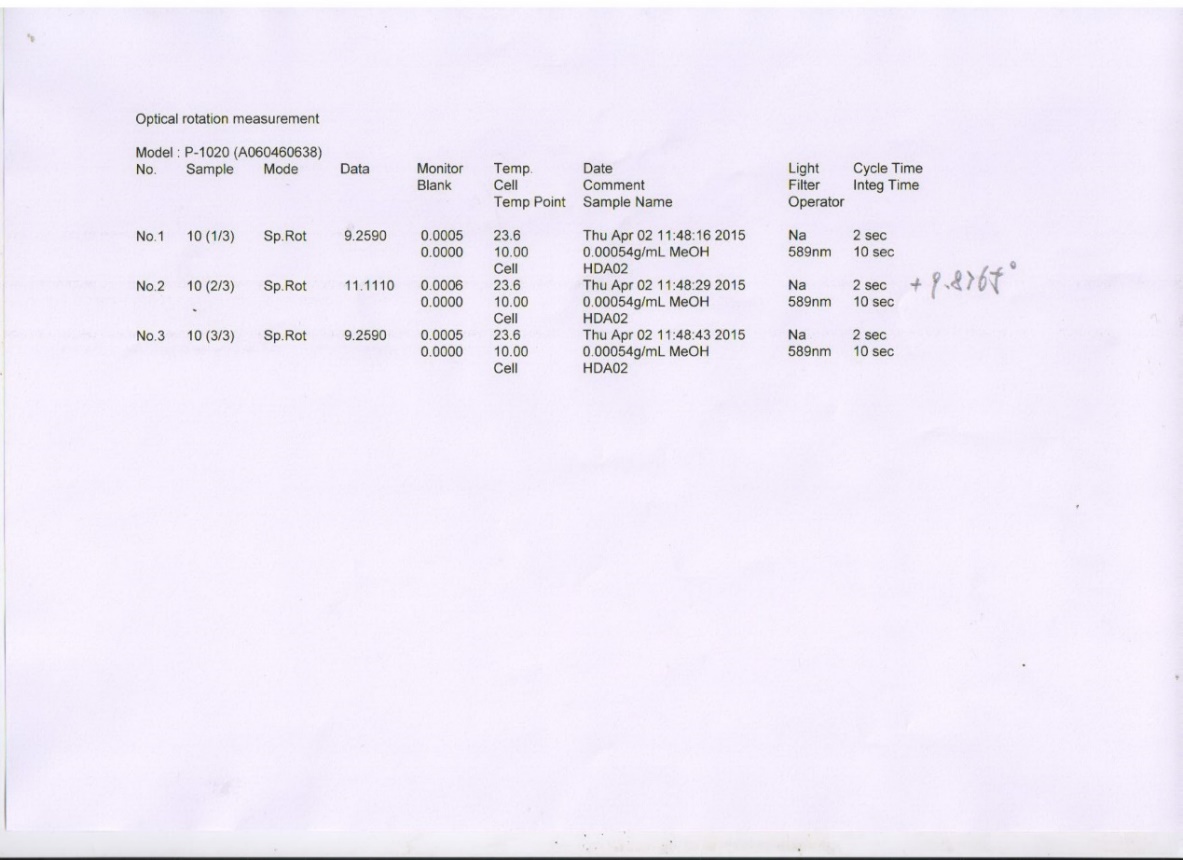


S1.11 ^1^H NMR spectrum of (-)-Nitrone 17 (**2**) in CDCl_3_


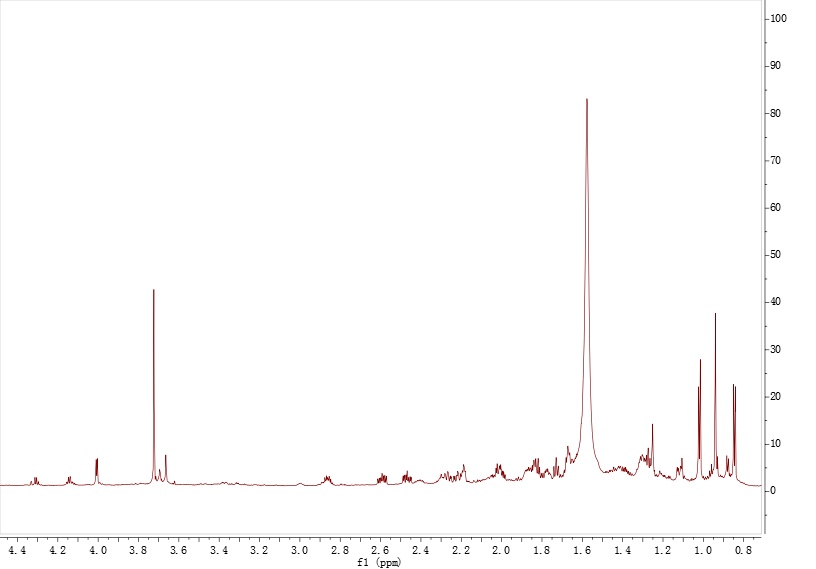


S1.12 ^13^C NMR spectrum of (-)-Nitrone 17 (**2**) in CDCl_3_


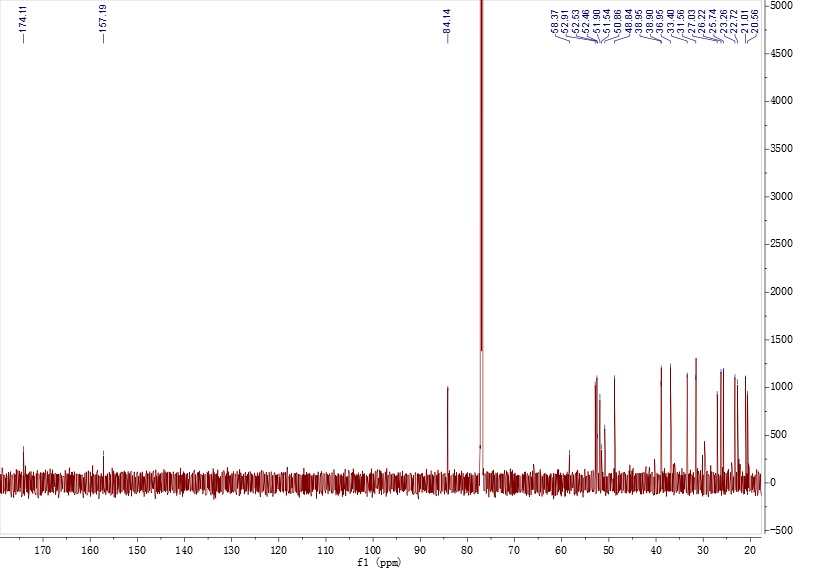


S1.13 ESIMS spectrums of (-)-Nitrone 17 (**2**)


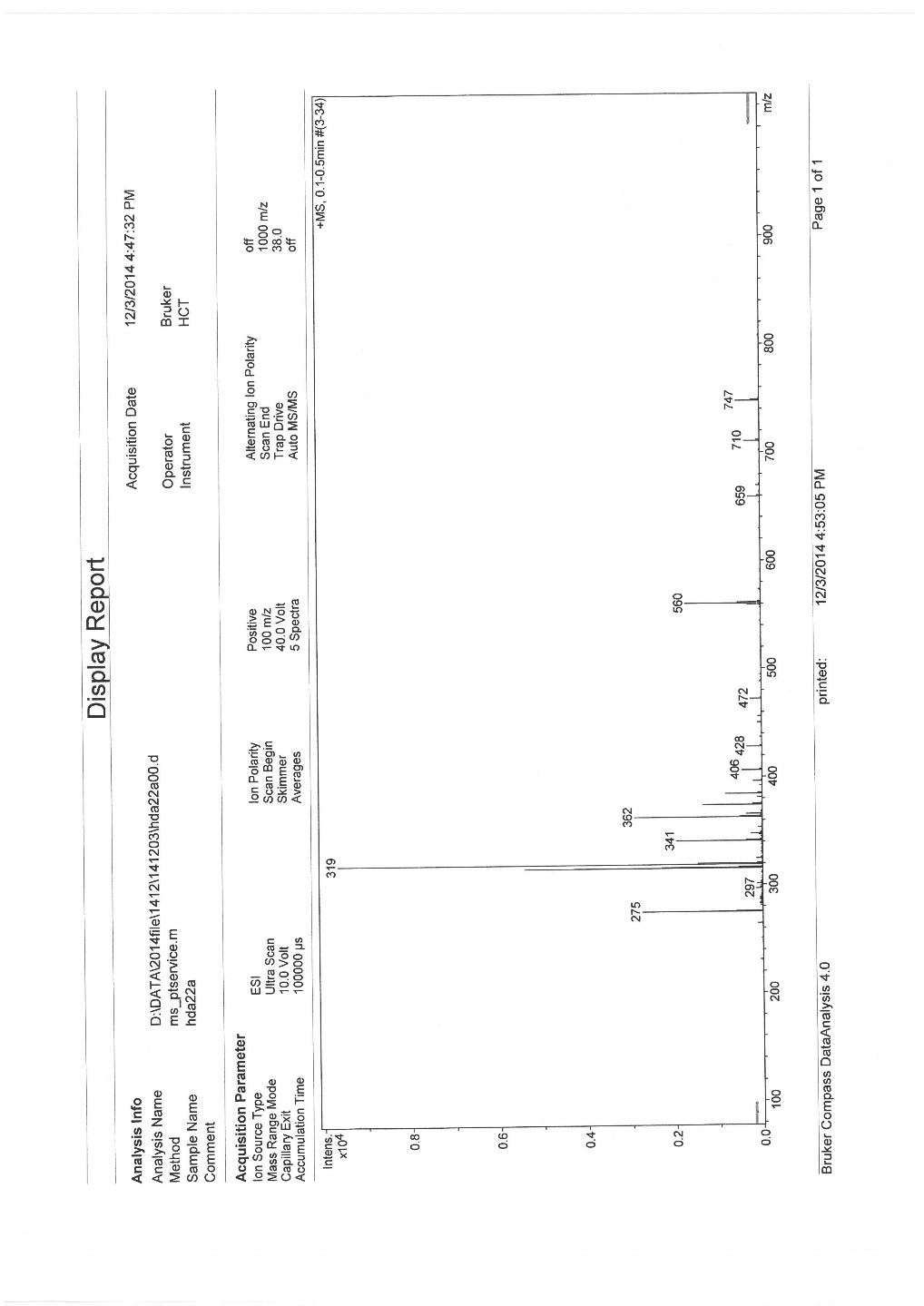


S1.14 Optical rotation spectrum of (-)-Nitrone 17 (**2**)


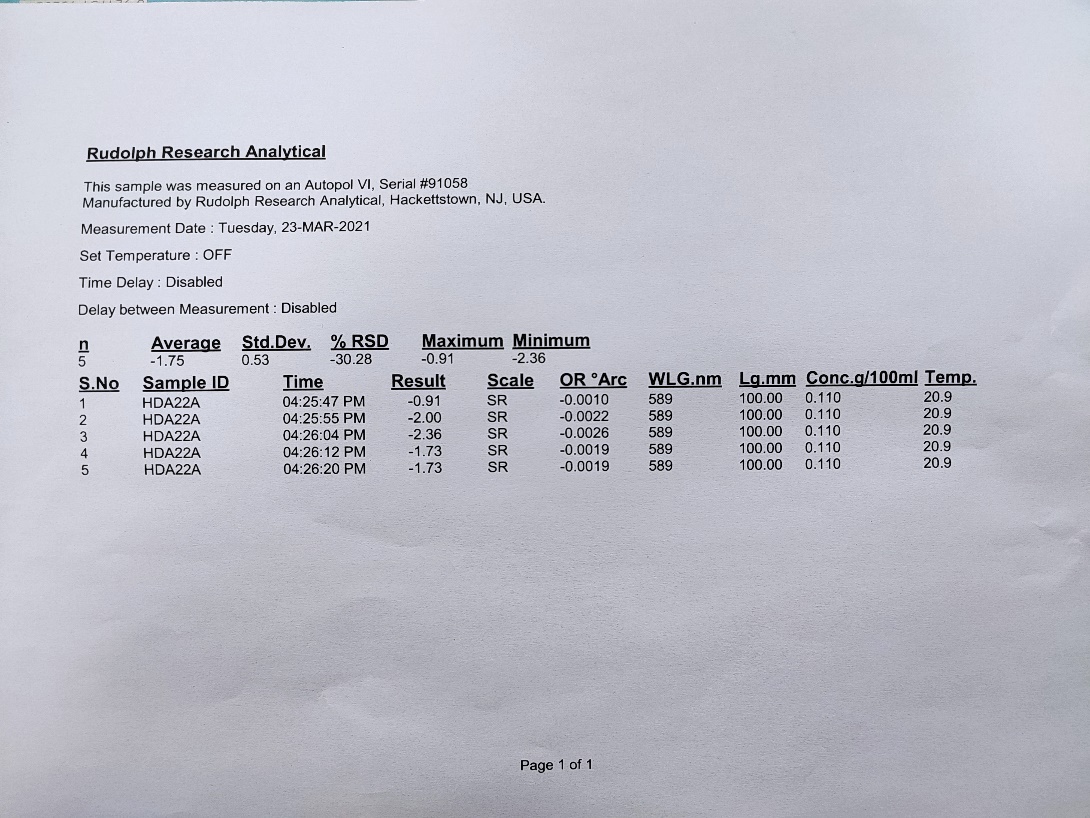

Supplement: Supplementary file 1 — Supplementary file1 (DOCX 2262 kb) [file 13659_2021_309_MOESM1_ESM.docx]
